# Serologic evidence of exposure to livestock-associated pathogens in free-ranging mountain tapir (*Tapirus pinchaque*) in Ecuador

**DOI:** 10.3389/fvets.2026.1811096

**Published:** 2026-07-15

**Authors:** Liza Dadone, Armando Castellanos, Barb Wolfe, Jorge Brito, Melchor Ascanta, Patricio Cruz, Budhan S. Pukazhenthi

**Affiliations:** 1 Cheyenne Mountain Zoo, Colorado Springs, CO, United States; 2 Giraffe Veterinary Services, Colorado Springs, CO, United States; 3 Andean Bear Foundation, Quito, Ecuador; 4 Instituto Nacional de Biodiversidad, Quito, Ecuador; 5 Department of Clinical Sciences, Colorado State University, Fort Collins, CO, United States; 6 Smithsonian National Zoo and Conservation Biology Institute, Front Royal, VA, United States

**Keywords:** Andes ecosystems, livestock-associated pathogens, one health, serology, *Tapirus pinchaque*, wildlife disease surveillance

## Abstract

Mountain tapir (*Tapirus pinchaque*) are endangered Andean ungulates for which infectious disease exposure in free-ranging populations remains poorly characterized. Between 2016 and 2018, serum samples were collected during field anesthesia events conducted for conservation management purposes within Cayambe-Coca National Park, Ecuador, and tested for selected bacterial and viral pathogens of livestock and zoonotic relevance, including *Brucella* spp., *Leptospira* spp., bovine viral diarrhea virus (BVDV), bovine herpesvirus 1 (BoHV-1), vesicular stomatitis virus (VSV), as well as Eastern and Western equine encephalitis viruses (EEEV, WEEV). Nine serum submissions representing eight individuals were evaluated using microscopic agglutination testing (MAT) for *Leptospira* spp., fluorescence polarization assay (FPA) with confirmatory complement fixation (CF) and repeat FPA testing at the U.S. National Veterinary Services Laboratories (NVSL) for *Brucella* spp., plaque reduction neutralization testing (PRNT) for EEEV and WEEV, and serum neutralization (SN) assays for BVDV, BoHV-1, and VSV. Most samples were seronegative for all tested pathogens (6/8 individuals; 75%), with all samples negative for antibodies to EEEV, WEEV, BVDV, BoHV-1, and VSV. However, one individual tested positive for *Brucella* antibodies, and one demonstrated antibodies to multiple *Leptospira* serovars. These findings provide baseline serologic data and indicate that exposure to pathogens associated with livestock, wildlife, and shared environments can occur within Andean landscapes that include both protected areas and adjacent human-use zones. Detection of *Brucella* antibodies, a pathogen of particular zoonotic and reproductive significance that may circulate in livestock and wildlife, highlights the importance of integrated pathogen surveillance across wildlife, livestock, and environmental interfaces. Serologic results indicate prior exposure rather than active infection or clinical disease. Continued integrated surveillance may improve understanding of pathogen circulation, exposure sources, and One Health risks in Andean ecosystems.

## Introduction

The genus *Tapirus* includes four extant species: *Tapirus terrestris* (lowland tapir), *Tapirus bairdii* (Baird's tapir), and *Tapirus pinchaque* (mountain tapir), distributed across Central and South America, as well as *Tapirus indicus* (Malayan tapir), which occurs in Southeast Asia (1). The mountain tapir is listed as endangered by the IUCN Red List, is the most threatened of the four extant tapir species and has the smallest geographic distribution, restricted to high-elevation Andean habitats of Colombia, Ecuador, and Peru (2). Their populations continue to decline due to habitat loss, fragmentation, and hunting pressure. Increasing landscape fragmentation can isolate populations genetically, reduce demographic resilience, and heighten vulnerability to additional stressors, including infectious diseases (1, 3).

Tapir are perissodactyls and may be susceptible to pathogens affecting other perissodactyls and domestic livestock, including horses and cattle. Several pathogens reported in tapir are also of zoonotic relevance, including *Brucella* spp. and *Leptospira* spp., highlighting the importance of characterizing pathogen exposure at livestock–wildlife interfaces (4–7). As large-bodied, wide-ranging herbivores, tapir may also serve as useful sentinels for pathogen circulation and broader ecosystem health stressors (8).

Despite their conservation status, relatively little is known about infectious disease exposure in free-ranging mountain tapir. Much of the published tapir health literature is derived from studies in managed-care settings or other tapir species, and recent syntheses note that baseline health data from free-ranging populations remain limited for many tapir taxa, including *T. pinchaque* (3, 8). To date, only limited pathogen exposure data have been reported for mountain tapir, including a single case of *Babesia caballi* in a rescued semi-captive calf from near the Cayambe-Coca National Park (9). These gaps limit inference regarding pathogen exposure patterns and potential conservation impacts in free-ranging mountain tapir.

Available field studies in lowland tapir (*Tapirus terrestris*) demonstrate that exposure patterns can vary substantially by landscape and interface intensity, including reports of antibodies to *Leptospira* spp. and occasional exposure to viral agents such as equine encephalitis viruses and bovine herpesvirus, while other surveys have found uniformly negative results for selected livestock-associated pathogens (10, 11). Additional reports in other tapir species, including *Tapirus bairdii*, have documented exposure to a broader range of infectious agents, such as hemoplasmas, piroplasmids, *Borrelia* spp., and Anaplasmataceae agents, highlighting the diversity of pathogens reported in tapirs across different ecological contexts (3, 8, 12–15).

In Ecuador, livestock production (particularly cattle) is widespread across Andean landscapes, including areas adjacent to and in some cases overlapping with protected areas. Endemic livestock pathogens include *Brucella* spp., *Leptospira* spp., bovine viral diarrhea virus (BVDV), and bovine herpesvirus 1 (BoHV-1). National and regional studies have documented substantial seroprevalence of these pathogens in Ecuadorian cattle populations (16–19). Leptospirosis is also increasingly recognized in Ecuador as a persistent, under-diagnosed zoonosis with ongoing transmission in multiple regions (20). Recent outbreak investigations further emphasize the value of One Health-aligned surveillance approaches that integrate human, livestock, and wildlife data streams (21). In these systems, indirect environmental exposure pathways may facilitate pathogen transmission between livestock and wildlife where species share water sources or overlapping habitats.

Protected areas such as Cayambe-Coca National Park play a critical role in conserving remaining mountain tapir populations. However, protected area boundaries do not necessarily prevent disease transmission where domestic animals and wildlife share landscapes. Livestock may enter protected areas for grazing, and wide-ranging wildlife species may move beyond park boundaries into adjacent agricultural landscapes. In this context, infectious diseases associated with livestock may represent an additional conservation consideration (7, 22).

The objective of this study was to describe serologic findings obtained opportunistically during field anesthesia events from free-ranging mountain tapir sampled within Cayambe-Coca National Park, Ecuador. By documenting exposure to selected bacterial and viral pathogens of livestock and zoonotic relevance, this study aims to provide baseline health data for an endangered species and inform future wildlife health surveillance and conservation planning.

## Materials and methods

### Ethical approvals and permits

Field captures, sample collection, and transport of biological materials were authorized by the Ecuadorian Ministerio del Ambiente y Energía (permit numbers 003-2015-AD-RIC-FAU-DPAP-MA and 004-FAU-2018-DPAP-MA). Samples were exported to the Smithsonian's National Zoo and Conservation Biology Institute by INABIO for analysis.

The samples collected in 2018 was obtained under a protocol approved by the Smithsonian Institutional Animal Care and Use Committee (SI_IACUC #18-03). Export and import of biological samples were conducted under CITES export permit 18EC000008/VS and CITES import permit 17US700310/9.

### Study population, capture, anesthesia, and sampling

Free-ranging mountain tapir were sampled in Cayambe-Coca National Park during anesthesia events conducted as part of ongoing conservation field activities (e.g., health assessments and radio or GPS collar placement), rather than targeted disease surveillance. Serum samples were collected opportunistically between October 2016 and March 2018. Exact ages were not known for these free-ranging individuals; animals were assessed in the field as adults based on body size and morphology.

Tapir were located by identifying fresh tracks with the assistance of experienced Andean trackers, with trained tracking dogs used to minimize search time. Once a tapir was visually located and approached, it was briefly manually restrained using lassos to facilitate safe dart placement and to limit movement during anesthetic delivery.

Anesthesia was induced using ketamine hydrochloride (400–500 mg; ~2.8 mg/kg; Ketaset, Zoetis, LLC, Kalamazoo, MI, USA), xylazine hydrochloride (400–500 mg; ~2.8 mg/kg; AnaSed, Cronus Pharma, East Brunswick, NJ, USA), and butorphanol tartrate (10–30 mg; 0.05–0.15 mg/kg; Torbugesic, Zoetis LLC, Kalamazoo, MI, USA) delivered in a single dart using a CO_2_-powered dart projector (Dan-Inject JM Standard, Dan-Inject ApS, Børkop, Denmark), consistent with previously published protocols for free-ranging mountain tapir and other large neotropical mammals (23, 24).

Physiological parameters including heart rate, respiratory rate, and body temperature were monitored throughout anesthesia. During anesthesia, animals received supportive care including vitamin E (500 mg IM, Vitamin E-300; AgriLabs, Inc., St. Joseph, MO, USA), flunixin meglumine (500 mg IM; Banamine, Merck Animal Health, Rahway, NJ, USA), and intravenous fluids as needed (Lactated Ringer's Injection, USP; ICU Medical, San Clemente, CA, USA). Supplemental ketamine (50–100 mg IV or IM) was administered as needed to maintain an adequate depth of anesthesia.

Anesthesia was reversed following completion of sampling procedures and prior to prolonged handling, transport of samples, or departure from the capture site. Following procedures, xylazine was antagonized using either yohimbine hydrochloride (40–50 mg IM; Musechem, Paoli, PA, USA) or atipamezole (Antisedan, Zoetis LLC, Kalamazoo, MI, USA). Butorphanol was antagonized using naltrexone (150 mg IM; Trexonil, Wildlife Pharmaceuticals, White River, South Africa). Animals were monitored through recovery until standing and coordinated prior to release at the capture site. No anesthetic-related complications were observed.

Blood samples were collected by venipuncture from the medial saphenous or jugular veins and transferred into serum separator tubes. Samples were transported on ice pack in a cooler to the field laboratory, centrifuged within approximately 3 h (1,000*g* for 10–15 min), and stored frozen in a liquid nitrogen dry-shipper. Upon reaching Quito, serum samples were transferred to a −80 °C freezer until shipment for diagnostic testing.

### Serologic testing

Frozen serum samples were submitted to the Cornell University Animal Health Diagnostic Center (Ithaca, NY, USA) for analysis, with confirmatory testing performed at the U.S. National Veterinary Services Laboratories (NVSL; Ames, IA, USA) for select assays.

Testing for *Brucella* spp. exposure was conducted using a fluorescence polarization assay (FPA), a competitive immunoassay that detects antibodies to smooth lipopolysaccharide antigens of *Brucella abortus* and *B. suis*. Results were interpreted based on laboratory-defined fluorescence polarization values (ΔmP), with values >20 considered positive, 10–20 considered suspect, and < 10 considered negative. Samples yielding positive or suspect results were submitted to NVSL for confirmatory testing using complement fixation (CF) and repeat FPA testing under regulatory diagnostic protocols. CF testing was performed using standardized *Brucella* antigen preparations with complement-mediated hemolysis as the endpoint, interpreted according to regulatory diagnostic criteria for livestock species. Appropriate positive and negative controls were included in all assays at the performing laboratories. Serologic testing does not differentiate infecting *Brucella* species due to cross-reactivity among *Brucella* spp. antigens.

Serologic testing for *Leptospira* spp. was performed using the microscopic agglutination (MAT) with a panel of reference serovars including *Leptospira interrogans* serovars Pomona, Hardjo, Icterohaemorrhagiae, Grippotyphosa, and Canicola. Testing utilized live antigen cultures maintained by the diagnostic laboratory at standard densities appropriate for MAT assays. Samples were initially screened at a 1:100 dilution, with titers < 1:100 interpreted as negative, consistent with USDA-recognized diagnostic thresholds. Positive samples were serially diluted in two-fold increments, and agglutination was assessed by comparison with control wells, with results reported as reciprocal antibody titers.

Plaque reduction neutralization testing (PRNT) was performed to detect neutralizing antibodies against Eastern equine encephalitis virus (EEEV) and Western equine encephalitis virus (WEEV). Testing was conducted using serial serum dilutions incubated with live virus in cell culture systems (e.g., mammalian cell lines maintained at the diagnostic laboratory), with reduction in plaque formation quantified relative to virus-only controls. Neutralization titers were determined based on ≥50% reduction in plaque formation (PRNT_50_), with titers reported as the highest dilution achieving this threshold (25). The minimum serum dilution tested was 1:20, with results reported as the reciprocal of the highest dilution producing viral neutralization.

Serum neutralization (SN) assays were conducted for bovine viral diarrhea virus (BVDV types 1 and 2), bovine herpesvirus 1 (BoHV-1), and vesicular stomatitis virus (VSV; Indiana serotype). Testing was performed using serial serum dilutions beginning at 1:8 in permissive cell culture systems, with cytopathic effect (CPE) assessed microscopically as the endpoint for determining viral inhibition, consistent with WOAH-referenced methodologies. Samples were reported as negative when no antibody was detected at the minimum dilution. In wildlife samples, heat inactivation may not be performed due to potential sample degradation, and low titers may be influenced by non-specific inhibitory factors, consistent with diagnostic laboratory reporting guidance.

All assays were performed at accredited diagnostic laboratories using validated protocols developed for domestic livestock species and included assay-specific cutoff thresholds and internal quality controls. Laboratory-defined criteria for positivity, negativity, and suspect classification were applied according to standard diagnostic protocols at the performing laboratories (Cornell University Animal Health Diagnostic Center and NVSL). Detailed assay parameters (e.g., reagent volumes, incubation conditions, and cell systems) followed established laboratory standard operating procedures consistent with published and WOAH-referenced diagnostic methodologies for livestock species. Assays were selected based on availability within accredited diagnostic laboratories and relevance to livestock-associated pathogens in the study region.

### Data analysis

Data were compiled and summarized using descriptive statistics. For each pathogen, the total number of samples tested and the number and proportion of samples meeting laboratory-defined criteria for positivity or regulatory classification were calculated.

A total of nine serum samples were obtained from eight individual mountain tapir. One individual was sampled on two occasions during a subsequent capture event for tracking collar replacement; these samples were retained and analyzed separately due to temporal separation of sampling events.

## Results

No anesthesia- or sampling-related complications (e.g., cardiorespiratory instability, prolonged recovery, or injury) were observed in any individual. Nine serum submissions representing eight individual mountain tapir were evaluated (Table 1). Most individuals (6/8; 75%) had no detectable antibodies to the selected panel of livestock-associated and zoonotic pathogens, based on laboratory-defined positivity thresholds as described in Section Materials and methods. One individual was sampled on two occasions; results from these temporally distinct sampling events were included in sample-level summaries but did not alter individual-level classification. All samples were seronegative for EEEV, WEEV, BVDV-1, BVDV-2, BoHV-1, and VSV.

**Table 1:** Summary of serologic testing results for free-ranging mountain tapir (*T. pinchaque*) sampled in Cayambe-Coca National Park, Ecuador (2016–2018).

Animal ID	Sample date	Sex	*Leptospira* MAT	*Brucella* FPA	EEEV PRNT (reciprocal titer)	WEEV PRNT	BVDV (T1/T2)	BoHV-1	VSV
Animal 1	10/17/2016	Male	Negative	Negative	Negative	Negative	Negative/Negative	Negative	Negative
Animal 2	10/18/2016	Male	Negative	**Positive** ^**a**^	Negative	Negative	Negative/Negative	Negative	Negative
Animal 4	10/23/2016	Female (pregnant)^b^	Negative	Negative	Negative	Negative	Negative/Negative	Negative	Negative
Animal 5	03/18/2018	Female	**Positive** ^**c**^	Inconclusive^d^	Negative	Negative	Negative/Negative	Negative	Negative
Animal 6	10/18/2016	Female	Negative	Negative	Negative	Negative	Negative/Negative	Negative	Negative
Animal 7^e^	10/26/2016	Female	Negative	Inconclusive^d^	QNS	Negative	Negative/Negative	Negative	Negative
Animal 7^e^	03/15/2018	Female	Negative	Negative	Negative	Negative	Negative/Negative	Negative	Negative
Animal 9	11/30/2016	Female	Negative	Negative	Negative	Negative	Negative/Negative	Negative	Negative
Animal 11	03/18/2018	Female	Negative	Negative	Negative	Negative	Negative/Negative	Negative	Negative
Summary	**—**	**2 male, 6 female**	**1/8 (12.5%) positive**	**1/8 (12.5%) positive**	**0/8**	**0/8**	**0/8**	**0/8**	**0/8**

Results are presented as qualitative interpretations (positive/negative/inconclusive) based on assay-specific thresholds described in the Section Materials and methods.

MAT, microscopic agglutination test; FPA, fluorescence polarization assay; PRNT, plaque reduction neutralization test; QNS, quantity not sufficient (this sample was excluded from assay-specific summary calculations where applicable); EEEV, Eastern equine encephalitis virus; WEEV, Western equine encephalitis virus; BVDV, bovine viral diarrhea virus; BoHV-1, bovine herpesvirus 1; VSV, vesicular stomatitis virus.

^a^Animal 2: Brucella FPA positive; confirmatory testing was performed under regulatory diagnostic protocols at the U.S. National Veterinary Services Laboratories (NVSL) and was consistent with regulatory classification of exposure.

^b^Pregnancy status was identified based on field assessment at the time of capture. Anesthesia was performed as part of ongoing conservation activities under established veterinary protocols, and no complications were observed.

^c^Leptospira MAT titers for Animal 5: Grippotyphosa (1:800); Pomona (1:200); Canicola (1:200); Icterohaemorrhagiae (1:100); Hardjo (< 1:100).

^d^Inconclusive Brucella FPA results reflect values falling within the laboratory-defined indeterminate range based on assay-specific cutoff thresholds. Samples with insufficient volume for complete testing were recorded as QNS (quantity not sufficient) and are reported accordingly.

^e^Animal 7 was sampled on two occasions (10/26/2016 and 3/15/2018); results are presented separately due to temporal separation of sampling events.

Negative results for PRNT and SN assays indicate no detectable antibody at the minimum assay dilution (e.g., < 1:10 for PRNT, < 1:8 for SN), according to assay-specific laboratory-defined thresholds described in Section Materials and methods.

Several assays were not validated for use in tapir; results are therefore interpreted descriptively according to laboratory reporting criteria.

Exact ages were not known for these free-ranging individuals; however, all animals were assessed in the field as adults based on body size and morphology. Summary represents an overview of results including percentages of animals that tested positive via serology.

Serologic evidence of prior exposure was identified in two of eight individuals (25%), including antibodies to *Leptospira* spp. and *Brucella* spp. One tapir demonstrated antibodies to multiple *Leptospira* serovars by microscopic agglutination testing (MAT), with reciprocal titers of 1:800 to serovar Grippotyphosa, 1:200 to serovars Pomona and Canicola, and 1:100 to serovar Icterohaemorrhagiae; antibodies to serovar Hardjo were below the laboratory detection threshold (< 1:100) and were therefore classified as seronegative according to assay-specific criteria.

A second individual tested positive for *Brucella* antibodies by fluorescence polarization assay (FPA), with confirmatory testing performed under regulatory diagnostic protocols at the National Veterinary Services Laboratories, including CF consistent with regulatory classification of exposure. Serologic testing does not allow determination of the infecting *Brucella* species due to cross-reactivity among *Brucella* spp. antigens.

No clinical abnormalities consistent with infectious disease were observed in any seropositive individuals at the time of capture.

## Discussion

### Serologic findings in free-ranging mountain tapir

This study provides baseline serologic data from free-ranging mountain tapir sampled within a protected Andean ecosystem, where empirical health data remain limited. Most individuals demonstrated no detectable antibodies to the pathogens evaluated. This pattern may reflect limited exposure, temporal variation in antibody persistence, or reduced assay sensitivity in a non-validated species. Comparable serologic surveys in *T. terrestris* have reported variable exposure patterns depending on landscape context, including both seropositive and uniformly seronegative populations for similar pathogen panels (10, 11), supporting the interpretation that exposure risk is highly context-dependent, consistent with patterns reported in other tapir species.

Serologic evidence of prior exposure was identified in two individuals (25%), including antibodies to *Brucella* spp. and *Leptospira* spp. These serologic findings indicate that exposure to pathogens associated with livestock, wildlife, and shared environmental sources can occur within landscapes that include both protected areas and adjacent human-use zones. The source of exposure cannot be determined from serology alone, and the detected antibodies should not be interpreted as evidence that transmission originated exclusively from livestock. Importantly, serologic results indicate prior exposure rather than active infection, pathogen shedding, or clinical disease, and no clinical abnormalities consistent with infectious disease were observed at the time of sampling.

### Brucella exposure and conservation relevance

Detection of *Brucella* antibodies is notable given the endangered status, low reproductive rate, and fragmented distribution of mountain tapir. The positive result was confirmed using regulatory diagnostic protocols at the National Veterinary Services Laboratories, supporting true exposure. However, serologic evidence alone does not confirm active infection, pathogen shedding, or clinical disease.

Brucellosis is well documented in livestock and wildlife globally and is associated with reproductive losses in many host species (26, 27). Although clinical brucellosis has not been described in tapir, the life history characteristics of mountain tapir, including long gestation and low reproductive output, suggest that even sporadic reproductive impacts could be biologically significant at the population level. The epidemiologic and clinical implications of *Brucella* exposure in *T. pinchaque* remain unknown.

In cattle, *Brucella abortus* is associated with late-term abortion, retained placenta, and infertility (28).

In Ecuador, including Andean regions overlapping the present study area, exposure to *Brucella canis* has been documented in wild canids, including Andean foxes (*Lycalopex culpaeus*), supporting the plausibility of environmental or indirect exposure pathways for wildlife species sharing livestock-dominated landscapes (29, 30).

### *Leptospira* exposure and environmental context

Serologic evidence of *Leptospira* exposure has been reported in other tapir species and is generally considered subclinical (10, 11). The seropositive individual in this study demonstrated reactivity to multiple *Leptospira* serovars, a pattern commonly associated with cross-reactivity inherent to MAT rather than definitive identification of the infecting serovar. Confirmation of infecting strains typically requires direct detection and molecular characterization (31, 32).

Leptospira antibodies have also been documented in Andean bear (*Tremarctos ornatus*) and free-ranging Andean fox (*Lycalopex culpaeus*) in Ecuador, including Andean ecosystems comparable to the present study area, supporting the environmental circulation of *Leptospira* spp. (29, 33). Reported serovars in these species include Pomona and Icterohaemorrhagiae, which overlap with those identified in the tapir in this study, supporting shared environmental exposure pathways across wildlife and livestock. Reported *Leptospira* serovars in Ecuadorian cattle include Pomona, Hardjo, and Icterohaemorrhagiae (20, 21), further supporting environmental circulation of these serovars within shared landscapes.

### Wildlife-livestock interface and transmission pathways

The pathogens identified in this study are consistent with well-known transmission pathways at wildlife-livestock interfaces, including environmental contamination with urine (*Leptospira* spp.) and exposure to infected reproductive tissues (*Brucella* spp.) (26, 34) in shared landscapes. These transmission pathways do not require direct contact between wildlife and livestock and may occur where species share water sources or overlapping landscapes (7).

Although all animals were sampled within Cayambe-Coca National Park, protected area boundaries do not fully isolate wildlife from surrounding land-use practices. Livestock grazing may occur within or adjacent to protected areas, and wide-ranging wildlife may move beyond park boundaries into surrounding agricultural landscapes (7, 22). In this context, exposure to livestock-associated pathogens may occur even in protected populations. However, *Brucella* spp. and *Leptospira* spp. may also circulate among wildlife hosts or persist in shared environmental sources. Therefore, although the study landscape includes livestock and human-use interfaces, the serologic results do not identify livestock as the definitive source of exposure and do not exclude wildlife-associated or environmentally maintained transmission pathways.

Habitat fragmentation compounds these risks by reducing genetic and demographic resilience. When combined with opportunistic hunting, even low-frequency disease exposure could contribute cumulatively to population decline and increase extinction risk over time (1, 3).

### One Health and conservation implications

*Brucella* spp. and *Leptospira* spp. are of zoonotic concern (26, 27), and detection of antibodies in free-ranging wildlife highlights the importance of appropriate biosafety practices during wildlife capture and handling (4, 6). In regions where wildlife may be hunted, potential human exposure could also occur through handling of carcasses or consumption of improperly cooked meat, representing an additional zoonotic interface beyond livestock contact (35–37). These serologic findings support integration of wildlife health surveillance within broader public health and conservation frameworks.

Importantly, these serologic findings do not imply reservoir status or transmission directionality. Exposure may reflect indirect contact with pathogens circulating in the broader environment through shared water sources, contaminated substrates, or vectors. Wildlife health surveillance should therefore be viewed as complementary to, rather than a substitute for, robust livestock health programs.

### Study limitations

This study was based on opportunistic sampling of a small number of individuals, with most animals sampled at a single point in time. One individual was sampled on two occasions, with consistent negative results across timepoints. Serologic assays used were developed for domestic species and have not been validated for use in *T. pinchaque*, which may influence sensitivity and specificity. In addition, neutralization assays in wildlife samples may be affected by non-specific inhibitory factors or lack of heat inactivation, which can influence interpretation of low antibody titers (25).

Serologic testing detects prior exposure and does not distinguish between past infection, cross-reactivity, or current infection status. Serology alone cannot determine the source of exposure or direction of transmission. Attribution of exposure to livestock, wildlife, or shared environmental sources would require additional diagnostics, such as direct pathogen detection, molecular characterization, and coordinated sampling of domestic animals, wildlife, and environmental reservoirs. These limitations restrict inference regarding prevalence, transmission dynamics, and clinical significance.

## Conclusions

This study provides baseline serologic data from free-ranging mountain tapir in a protected Andean ecosystem and documents evidence of prior exposure to selected pathogens of livestock, wildlife, and zoonotic relevance. Although most individuals were seronegative and no clinical illness was observed at the time of sampling, detection of antibodies to *Brucella* spp. and *Leptospira* spp. indicates that pathogen exposure occurs within landscapes that include both protected areas and adjacent human-use zones.

Given the endangered status of the mountain tapir, its fragmented distribution, and low reproductive rate, even sporadic pathogen exposure may be relevant to long-term population viability. These findings support continued, integrated surveillance across wildlife, livestock, and environmental interfaces and highlight the value of linking wildlife health monitoring with livestock disease management and broader ecosystem health programs in Andean landscapes. Such approaches may improve understanding of environmental pathogen exposure and inform conservation strategies for mountain tapir and other native species.

## Data Availability

The original contributions presented in the study are included in the article/supplementary material, further inquiries can be directed to the corresponding author.

## References

[1] Melletti M Reyna-Hurtado R Medici EP (eds.). Tapirs of the World: Ecology, Conservation and Management. Cham: Springer (2024). doi: 10.1007/978-3-031-65311-7

[2] Lizcano DJ Amanzo J Castellanos A Tapia A Lopez-Malaga CM. (2016). Tapirus pinchaque. The IUCN Red List of Threatened Species. International Union for Conservation of Nature, p. e.T21473A45173922. doi: 10.2305/IUCN.UK.2016-1.RLTS.T21473A45173922.en

[3] Mangini PR Medici EP Fernandes-Santos RC. Tapir health and conservation medicine. Integr Zool. (2012) 7:331–45. doi: 10.1111/j.1749-4877.2012.00323.x 23253365

[4] Daszak P Cunningham AA Hyatt AD. Emerging infectious diseases of wildlife—threats to biodiversity and human health. Science. (2000) 287:443–9. doi: 10.1126/science.287.5452.443 10642539

[5] Jones KE Patel NG Levy MA Storeygard A Balk D Gittleman JL . Global trends in emerging infectious diseases. Nature. (2008) 451:990–3. doi: 10.1038/nature06536 18288193 PMC5960580

[6] Karesh WB Dobson A Lloyd-Smith JO Lubroth J Dixon MA Bennett M . Ecology of zoonoses: natural and unnatural histories. Lancet. (2012) 380:1936–45. doi: 10.1016/S0140-6736(12)61678-X 23200502 PMC7138068

[7] Wiethoelter AK Beltrán-Alcrudo D Kock R Mor SM. Global trends in infectious diseases at wildlife–livestock interface. Proc Natl Acad Sci USA. (2015) 112:9662–7. doi: 10.1073/pnas.1422741112 26195733 PMC4534210

[8] Ordonneau D Fernandes-Santos RC Zimmerman D Pukazhenthi B Rojas-Jumenez J Flores JP . Tapir health. In:Melletti M Reyna-Hurtado R Medici EP, editors. Tapirs of the World: Ecology, Conservation and Management. Cham: Springer (2024), p. 167–206.

[9] Castellanos A. First report of positive serological response to the hemoparasite, *Babesia caballi*, in mountain tapir. Tapir Conserv. (2013) 22:9. Available online at: http://www.tapirconservation.org/index.php/tc/article/view/147

[10] Furtado MM Jácomo AT Kashivakura CK Tôrres NM Marvulo MF Ragozo AM . Serologic survey for selected infectious diseases in free-ranging Brazilian tapirs (*Tapirus terrestris*) in the cerrado of central Brazil. J Zoo Wildl Med. (2010) 41:133–6. doi: 10.1638/2007-0087.1 20722266

[11] Medici EP Mangini PR Fernandes-Santos RC. Health assessment of wild lowland tapir (*Tapirus terrestris*) populations in the Atlantic Forest and Pantanal biomes, Brazil (1996–2012). J Wildl Dis. (2014) 50:817–28. doi: 10.7589/2014-02-029 25105810

[12] Mongruel ACB Medici EP da Costa Canena A Calchi AC; Perles L; Rodrigues BCB . *Theileria terrestris* nov. sp: A novel Theileria in lowland tapirs (Tapirus terrestris) from two different biomes in Brazil. Microorganisms. (2022) 10:2319. doi: 10.3390/microorganisms10122319 36557572 PMC9784709

[13] Mongruel ACB Medici EP da Costa Canena A Calchi AC Machado RZ André MR. Expanding the universe of hemoplasmas: multi-locus sequencing reveals putative novel hemoplasmas in lowland tapirs (*Tapirus terrestris*), the largest land mammals in Brazil. Microorganisms. (2022) 10:614. doi: 10.3390/microorganisms10030614 35336189 PMC8950906

[14] Mongruel ACB Medici EP da Costa Canena A Machado RZ Clay K Labruna MB . First molecular detection of Borrelia sp. in tapirs (*Tapirus terrestris*). Vet Res Commun. (2024) 48:2767–74. doi: 10.1007/s11259-024-10406-z 38713407

[15] Mongruel ACB Medici EP da Costa Canena A Cordova ASA Freitas das Neves L Franco EO . Molecular survey of vector-borne agents in lowland tapirs (*Tapirus terrestris*) from Brazil reveals a new *Anaplasma* genotype. Acta Trop. (2024) 260:107476. doi: 10.1016/j.actatropica.2024.107476 39608660

[16] Carbonero A Saa LR Jara DV García-Bocanegra I Arenas A Borge C . Seroprevalence and risk factors associated with bovine herpesvirus 1 infection in non-vaccinated dairy and dual-purpose cattle herds in Ecuador. Prev Vet Med. (2011) 100:84–8. doi: 10.1016/j.prevetmed.2011.03.006 21501883

[17] Saa LR Perea A García-Bocanegra I Arenas AJ Jara DV Ramos R . Seroprevalence and risk factors associated with bovine viral diarrhea virus (BVDV) infection in non-vaccinated dairy and dual purpose cattle herds in Ecuador. Trop Anim Health Prod. (2012) 44:645–9. doi: 10.1007/s11250-011-9948-4 21822791

[18] Ruano MP Burgos Macías DI Goicochea CAB Zambrano Aguayo MD Sandoval Valencia HP Falconi Flores MA . Seroprevalence and risk factors of bovine leptospirosis in the province of Manabí, Ecuador. Comp Immunol Microbiol Infect Dis. (2020) 72:101527. doi: 10.1016/j.cimid.2020.101527 32801110

[19] Garrido-Haro A Barrionuevo-Samaniego M Moreno-Caballeros P Burbano-Enriquez A Sánchez-Vázquez MJ Pompei J . Seroprevalence and risk factors related to bovine brucellosis in continental Ecuador. Pathogens. (2023) 12:1134. doi: 10.3390/pathogens12091134 37764942 PMC10536672

[20] Calvopiña M Romero-Alvarez D Vasconez E Valverde-Muñoz G Trueba G Garcia-Bereguiain MA . Leptospirosis in Ecuador: current status and future prospects. Trop Med Infect Dis. (2023) 8:202. doi: 10.3390/tropicalmed8040202 37104328 PMC10141158

[21] Orlando SA Mora-Jaramillo N Paredes-Núñez D Rodriguez-Pazmiño AS Carvajal E León Sosa A . Leptospirosis outbreak in Ecuador in 2023: a pilot study for surveillance from a One Health perspective. One Health. (2024) 19:100948. doi: 10.1016/j.onehlt.2024.100948 39717537 PMC11664413

[22] Manlove KR Sampson LM Borremans B Cassirer EF Miller RS Pepin KM . Epidemic growth rates and host movement patterns shape management performance for pathogen spillover at the wildlife–livestock interface. Philos Trans R Soc Lond B Biol Sci. (2019) 374:20180343. doi: 10.1098/rstb.2018.0343 31401952 PMC6711312

[23] Pukazhenthi B Richard R Mangini PR Zimmerman D. Tapirs. In: West G Heard D Caulkett N, editors. Zoo Animal and Wildlife Immobilization and Anesthesia, 3rd Edn. Hoboken, NJ: Wiley (2025), p. 977–90. doi: 10.1002/9781119539278.ch46

[24] Castellanos A Arias L Ascanta M Dadone L Pukazhenthi B Martínez E . Mamíferos grandes y medianos en Ecuador: recomendaciones para su captura y manejo. Mamm Aequat. (2026) 8:103–36. doi: 10.59763/mam.aeq.v8i1.133

[25] Weaver SC Reisen WK. Present and future arboviral threats. Antiviral Res. (2010) 85:328–45. doi: 10.1016/j.antiviral.2009.10.008 19857523 PMC2815176

[26] Corbel MJ. Brucellosis in Humans and Animals. Geneva: World Health Organization (2006).

[27] Godfroid J Garin-Bastuji B Saegerman C Blasco JM. Brucellosis in terrestrial wildlife. Rev Sci Tech. (2013) 32:27–42. doi: 10.20506/rst.32.1.2180 23837363

[28] World Organisation for Animal Health (WOAH) (2024). Infection with Brucella abortus, B. melitensis and B. suis. Paris: Terrestrial Animal Health Code. Available online at: https://www.woah.org/fileadmin/Home/eng/Health_standards/tahm/3.01.04_BRUCELLOSIS.pdf (Accessed January 24, 2026).

[29] Veintimilla N. Presencia de enfermedades parasitarias e infecciosas (Leptospirosis, distemper y brucelosis) en zorros andinos (Lycalopex culpaeus) que habitan en los páramos de la Hacienda Antisanilla (Pintag-Ecuador) (Undergraduate thesis). USFQ (2015). Available online at: https://repositorio.usfq.edu.ec/bitstream/23000/4211/1/113751.pdf (Accessed February 13, 2026).

[30] Castellanos A Yánez AE Arias L Castellanos F. First Report of Brucella canis in an Andean fox (Lycalopex culpaeus) in the Cotopaxi National Park, Ecuador. Technical Report. Quito (2020). Available online at: https://www.researchgate.net/publication/342693715_Brucella_canis_in_an_Andean_Fox_in_Ecuador (Accessed February 13, 2026).

[31] Chirathaworn C Inwattana R Poovorawan Y Suwancharoen D. Interpretation of microscopic agglutination test for leptospirosis diagnosis and seroprevalence. Asian Pac J Trop Biomed. (2014) 4:S162–4. doi: 10.12980/APJTB.4.2014C580 25183074 PMC4025277

[32] Guedes IB de Souza GO Castro JFP Cavalini MB de Souza Filho AF Heinemann MB. Usefulness of the ranking technique in the microscopic agglutination test (MAT) to predict the most likely infecting serogroup of *Leptospira*. *Front Vet Sci*. (2021) 8:654034. doi: 10.3389/fvets.2021.654034 PMC7965942 33748224

[33] Castellanos A Jackson D Arias L. Guidelines for the Rescue, Rehabilitation, Release, and Post-release Monitoring of Andean bears. Quito: Andean Bear Foundation (2016).

[34] Adler B de la Peña Moctezuma A. Leptospira and leptospirosis. Vet Microbiol. (2010) 140:287–96. doi: 10.1016/j.vetmic.2009.03.012 19345023

[35] Rhyan JC Spraker TR. Emergence of diseases from wildlife reservoirs. Vet Pathol. (2010) 47:34–9. doi: 10.1177/0300985809354466 20080482

[36] Karmacharya D Herrero-García G Luitel B Rajbhandari R Balseiro A. Shared infections at the wildlife–livestock interface and their impact on public health, economy, and biodiversity. Anim Front. (2024) 14:20–9. doi: 10.1093/af/vfad067 38369992 PMC10873012

[37] Díaz EA Arroyo G Sáenz C Mena L Barragán V. Leptospirosis in horses: Sentinels for a neglected zoonosis? A systematic review. Vet World. (2023) 16:2110–9. doi: 10.14202/vetworld.2023.2110-2119 38023277 PMC10668546

